# Supplementary material for: SpNeigh: spatial neighborhood and differential expression analysis for high-resolution spatial transcriptomics
Source: NAR Genom Bioinform. 2026 Apr 8;8(2):lqag039. doi: 10.1093/nargab/lqag039 (PMC13069690; doi:10.1093/nargab/lqag039)
Supplement: lqag039_Supplemental_File [file lqag039_supplemental_file.pdf]

Supplementary materials for "SpNeigh: spatial neighborhood and differential expression analysis for high-resolution spatial transcriptomics"

Jinming Cheng[1,2,*], Pierce Kah Hoe Chow[3,4], and Nan Liu[1,2,5,6,7,*]

30 January 2026

Contents

1 Supplementary Table and Figures **2**

[1]Centre for Biomedical Data Science, Duke-NUS Medical School, 169857, Singapore,
[2]Duke-NUS AI + Medical Sciences Initiative, Duke-NUS Medical School, 169857, Singapore,
[3]Surgery Academic-Clinical Program, Duke-NUS Medical School, 169857, Singapore,
[4]Department of Hepato-pancreato-biliary and Transplant Surgery, Singapore General Hospital and National Cancer Centre Singapore, 169610, Singapore,
[5]Pre-hospital & Emergency Research Centre, Health Services Research & Population Health, Duke-NUS Medical School, 169857, Singapore,
[6]NUS Artificial Intelligence Institute, National University of Singapore, 119077, Singapore,
[7]Department of Biostatistics and Bioinformatics, Duke University, 27710, NC, USA,
[*]Corresponding authors. Emails: jinming.cheng@outlook.com, jinming.cheng@duke-nus.edu.sg; liu.nan@duke-nus.edu.sg

1 Supplementary Table and Figures

Supplementary Table. S1: Manually merged SingleR cell type annotation for mouse brain.

New_Merged_CellType	Raw_SingleR_CellType
Hippocampus	CA1-ProS, CA2-IG-FC, CA3, DG, SUB-ProS, CT SUB
L2/3	L2 IT ENTl, L2 IT ENTm, L2/3 IT ENTl, L3 IT ENT, L2/3 IT CTX, L2/3 IT PPP, L2/3 IT RHP
L4/5/6	L4 RSP-ACA, L4/5 IT CTX, L5 IT CTX, L5 PPP, L5 PT CTX, NP PPP, NP SUB, L6 CT CTX, L6 IT CTX, L6 IT ENTl, L5/6 IT TPE-ENT, L5/6 NP CTX, Car3
L6b	L6b CTX, L6b/CT ENT
Astrocytes	Astro
Oligodendrocytes	Oligo
CGE	Lamp5, Sncg, Vip
MGE	Sst, Sst Chodl, Pvalb
Vascular	Endo, VLMC, SMC-Peri
Other	Meis2, CR, Micro-PVM

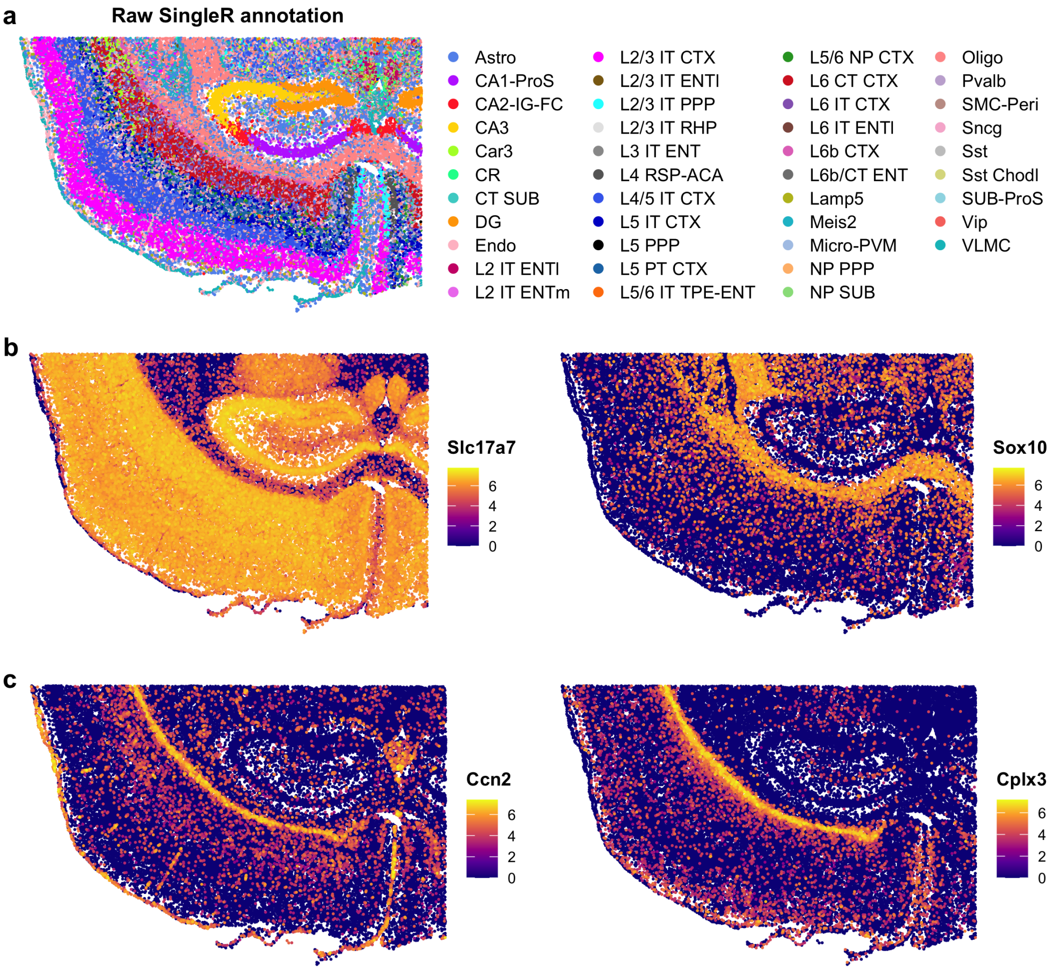

Supplementary Fig. S1: Cell type annotation and gene expression in mouse brain Xenium data. **a.** Spatial plot colored by cell-level annotations assigned using SingleR. **b.** Spatial expression of Slc17a7 and Sox10. Slc17a7 is a marker for excitatory neurons; Sox10 marks oligodendrocytes. **c.** Spatial expression of Ccn2 and Cplx3, genes enriched in boundary-associated neuronal populations.

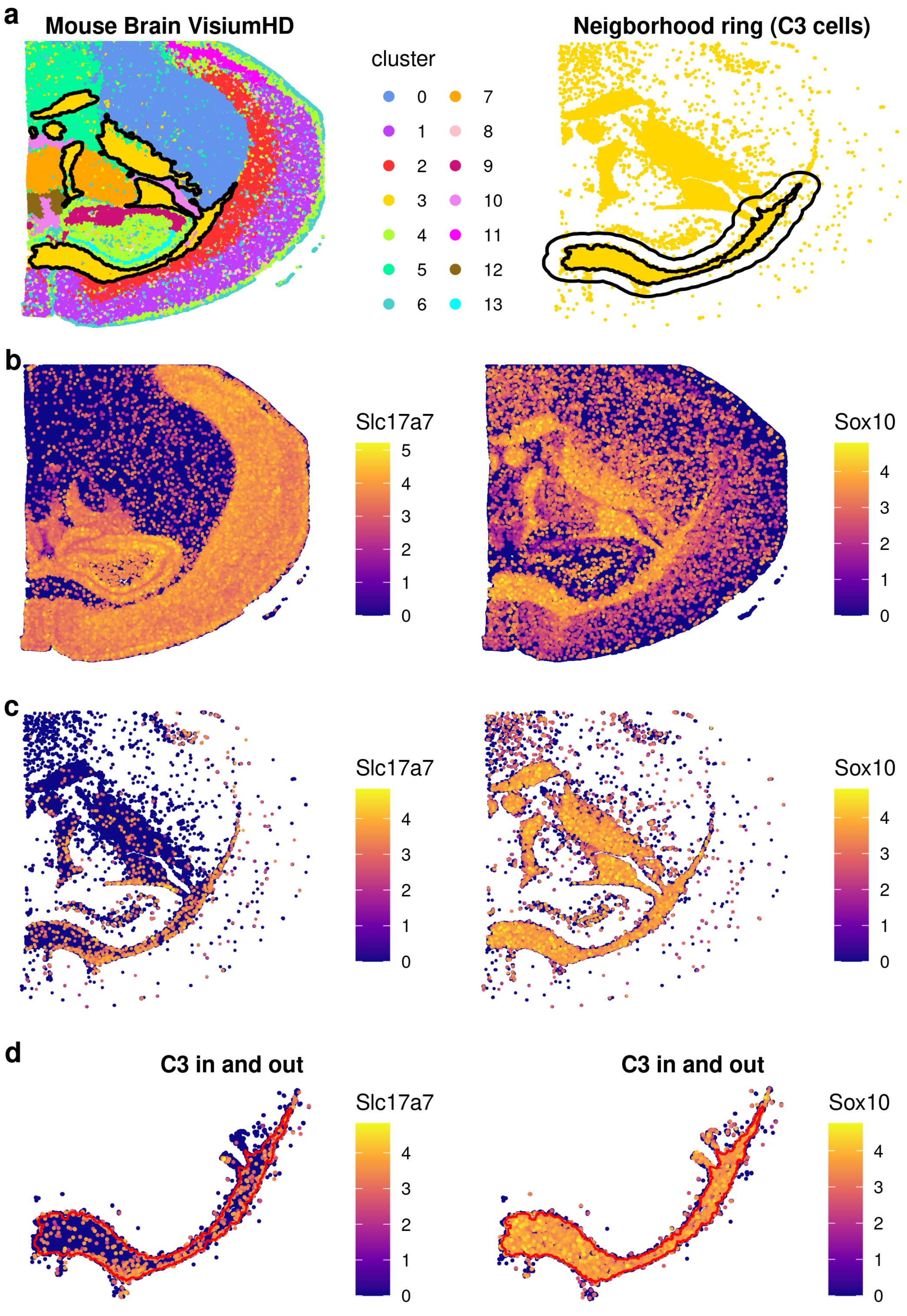

Supplementary Fig. S2: Spatial expression of Slc17a7 and Sox10 in mouse brain Visium HD data. **a.** Spatial map colored by clusters, highlighting cluster 3 (oligodendrocytes) and its primary neighborhood ring. Left: All clusters are shown with boundaries overlaid; cluster 3 cells are localized to white matter. Right: Cluster 3 cells are shown with their neighborhood ring relative to the primary boundary that separates white matter oligodendrocytes from cortical neurons. **b.** Spatial expression of Slc17a7 (neuronal marker) and Sox10 (oligodendrocyte marker) across all cells. **c.** Expression of Slc17a7 and Sox10 within cluster 3 cells. **d.** Expression of Slc17a7 and Sox10 in cluster 3 cells located within the primary boundary and its neighboring ring.

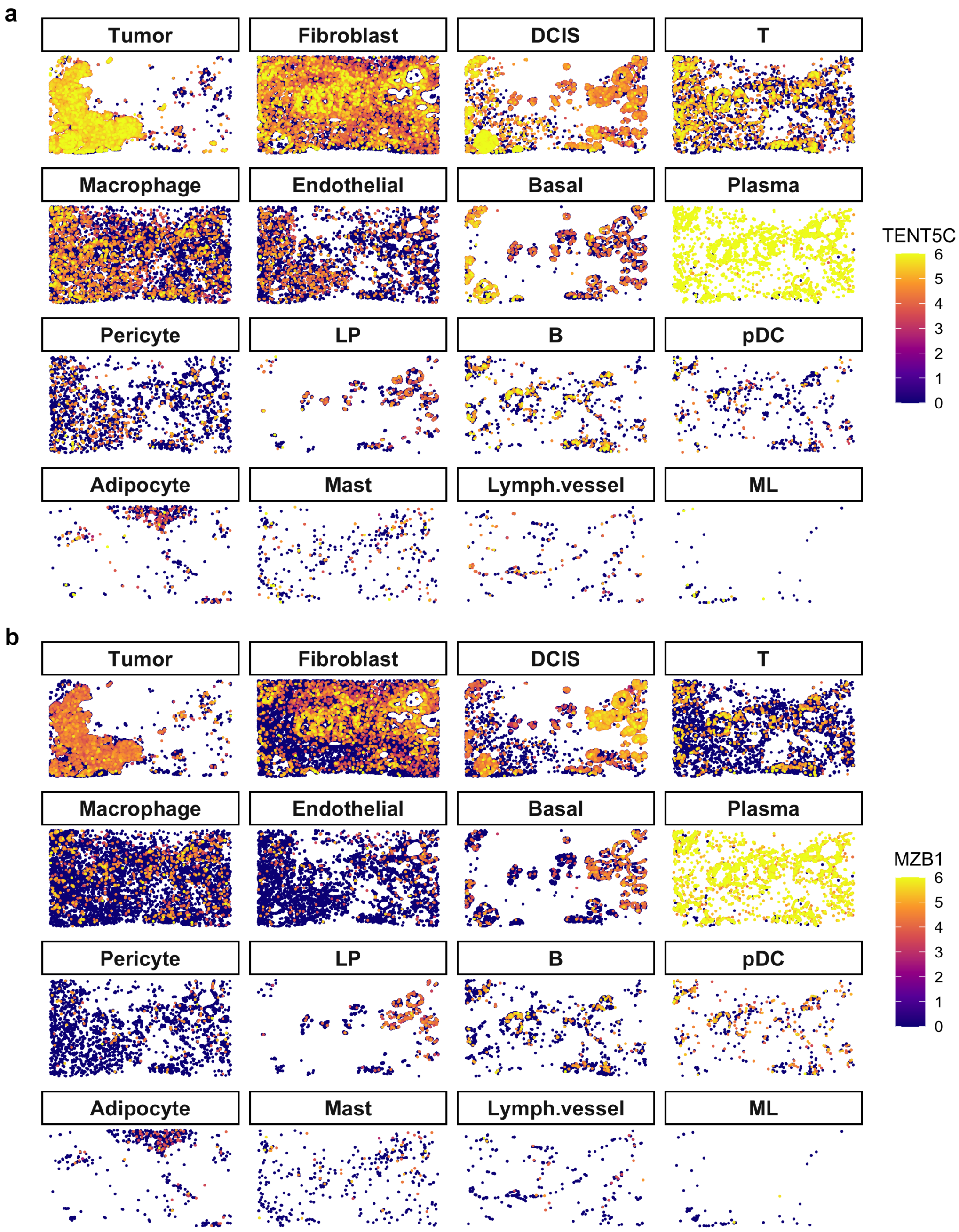

Supplementary Fig. S3: Expression of TENT5C and MZB1 across cell types in breast cancer Xenium data. **a.** Spatial expression of TENT5C across annotated cell types. **b.** Spatial expression of MZB1 across annotated cell types.

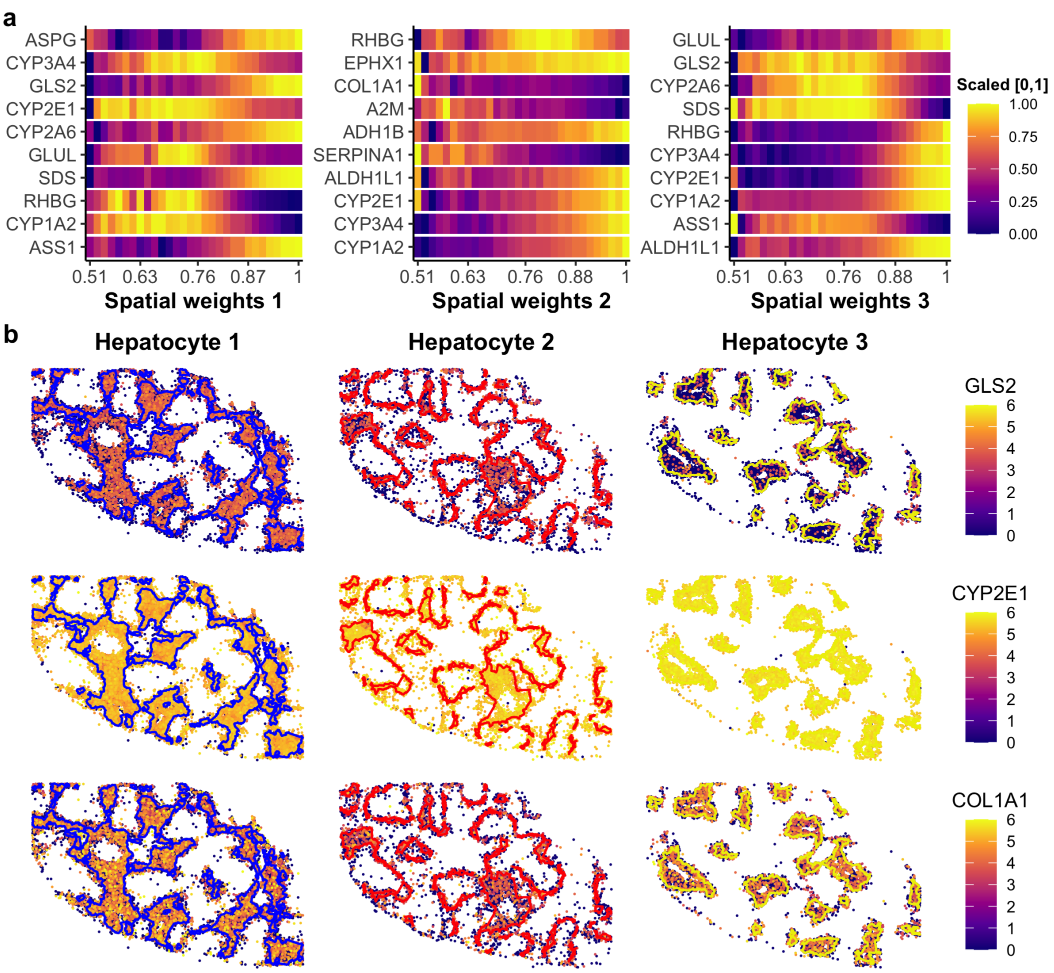

Supplementary Fig. S4: Spatial expression patterns of top spatially varying genes in liver hepatocytes. **a.** Heatmap showing the expression of the top 10 spatially differential genes along binned spatial weights for each hepatocyte population. Average expression values were rescaled to the range [0,1] to aid visualization. **b.** Spatial expression plots of GLS2, CYP2E1, and COL1A1 across the three hepatocyte populations. GLS2 is a periportal marker (hepatocyte 1), and CYP2E1 is a pericentral marker (hepatocyte 3); both show intermediate expression in mid-lobular hepatocytes (hepatocyte 2). COL1A1 shows lowest expression in hepatocyte 2. Blue, red, and yellow lines indicate the boundaries of hepatocyte populations 1, 2, and 3, respectively.
